# Real time monitoring of cold Ca^2+^ dependent transcription and its modulation by NCX inhibitors

**DOI:** 10.1038/s41598-022-22166-4

**Published:** 2022-10-15

**Authors:** Hsin-tzu Wang, Shiori Miyairi, Miho Kitamura, Kosuke Iizuka, Yoshimasa Asano, Takashi Yoshimura, Naohiro Kon

**Affiliations:** 1 Institute of Transformative Bio-Molecules (ITbM), Nagoya University, Furo-cho, Chikusa-ku, Nagoya, 464-8601 Japan; 2 Laboratory of Animal Integrative Physiology, Graduate School of Bioagricultural Sciences, Nagoya University, Furo-cho, Chikusa-ku, Nagoya, 464-8601 Japan; 3 Department of Biological Science, School of Science, The University of Tokyo, 7-3-1 Hongo, Bunkyo-ku, Tokyo, 113-0033 Japan; 4 Suntory Rising Stars Encouragement Program in Life Sciences (SunRiSE), 8-1-1 Seikadai, Seika-cho, Soraku-gun, Kyoto, 619-0284 Japan

**Keywords:** Biological techniques, Cell biology

## Abstract

Real-time monitoring of cellular temperature responses is an important technique in thermal biology and drug development. Recent study identified that Na^+^/Ca^2+^ exchanger (NCX)-dependent Ca^2+^ influx transduces cold signals to circadian clock in mammalian cultured cells. The finding raised an idea that cellular responses to the cold signals can be analyzed by monitoring of clock gene expression. We found that *Per1* and *Per2* were up-regulated after culture at 27 °C compared to 37 °C in Rat-1 fibroblasts. In order to monitor cold-Ca^2+^-dependent transcription in living cells, we developed a luciferase-based real-time reporting system by using *Per1* promoter*, Per2* promoter, Ca^2+^/cAMP-response elements (CRE) or NFAT-binding elements. We found that benzyloxyphenyl NCX inhibitor KB-R7943 and SN-6, but not SEA-0400 or YM-244769 inhibited the cold induction of *Per2*. Our study established a real-time monitoring system for cold Ca^2+^ signaling which can be applied to evaluation of drugs.

## Introduction

Temperature is one of the most important factors for maintenance of cellular homeostasis^1^. In response to changes of ambient temperature, gene expression levels are globally influenced to alter various cellular process including metabolic activities and ion transport activities^1,2^. In molecular mechanism of temperature signaling, temperature-sensitive ion channels expressed in neuronal cells, such as transient receptor potential (TRP) channels, have been intensively studied in animals^3^. Importantly, even in non-neuronal cells of animals, ambient temperature largely affects cellular physiology^3,4^. In addition, clear temperature responses were observed among fungi^5–7^, plants^8^ and bacteria^9^, while the TRP channels are not present in these organisms. These studies implicate an existence of basic mechanism conserved for temperature response in a wide variety of organisms.

Among various temperature responses, the circadian clock is of particular interest because of its unique property, *i.e.*, temperature-compensation of its period length^10,11^. Temperature compensation, observed in circadian clock of mammals, insects, fungi, plants and cyanobacteria, is a fundamental property of the cellular clock^12–15^. In mammals, the circadian rhythms are generated by transcriptional and translational feedback loops (TTFLs)^11^. Heterodimers of bHLH transcription factors CLOCK and BMAL1 bind to E-box DNA elements and activates thousands of genes including *Per* and *Cry* genes. The translated PERs bind to CRYs to inhibit the transcriptional activity of CLOCK-BMAL1. Thus, gene expression levels of *Per* genes show clear circadian rhythms^16^. Because most of the biochemical reactions in the TTFLs are slowed down by temperature decrease, it has been a mystery how circadian clock maintains its stable period length under different temperatures^17^.

Recently, we found that cytoplasmic Ca^2+^ signaling is activated by lowering temperature for compensation of the transcriptional oscillation^18^. In response to temperature decrease, Na^+^/Ca^2+^ exchanger (NCX) promotes Ca^2+^ influx, which activates Ca^2+^/calmodulin-dependent protein kinase II (CaMKII). CaMKII phosphorylates CLOCK to promote transcription of *Per1/2*^19^*,* and the transcriptional activation of clock genes accelerates oscillation of transcriptional feedback loops. The study clarified that the circadian clock is highly responsive to ambient temperature, and Ca^2+^ signaling transduces the temperature information to the TTFLs. Importantly, NCX-dependent cold Ca^2+^ signaling is functionally conserved among mammals, *Drosophila*, *Arabidopsis* and cyanobacteria, indicating that the cold Ca^2+^ signaling is a general temperature response inherited from a common ancestor of the organisms^18^. Based on the study, we thought that development of a real-time monitoring system for the temperature response of the molecular clock is an important technique to study the conserved temperature signaling in living cells.

In the present study, we found that the expression levels of *Per1* and *Per2* were up-regulated after cold exposure at 27 °C compared to 37 °C. The cold-induced transcriptional changes can be monitored by luciferase reporter driven by *Per1* and *Per2* promotor in the living Rat-1 fibroblasts. Importantly, we found that a Ca^2+^-dependent transcriptional reporter including Ca^2+^/cAMP response elements (CRE)^20^ or nuclear factor of activated T-cells (NFAT)^21^ binding elements, also showed temporal increase of bioluminescence levels after temperature shift from 37 to 27 °C. By using the cellular monitoring system, we investigated effects of various NCX inhibitors on the cold induction of *Per2*. We found that KB-R7943 and SN-6, but not SEA-0400 or YM-244769 dose-dependently inhibited the cold induction of *Per2*. Importantly, the actions on the *Per2* induction correlates with pharmacological actions on amplitude of the circadian rhythm especially at lower temperature. These results demonstrate that the real-time monitoring of *Per2* expression levels is a novel method for detecting temperature responses in living cells, and the system enable us to evaluate bioactivities of chemical compounds for drug development.

## Results

### Real-time monitoring of bioluminescence rhythms by *Per1*, *Per2* or *Bmal1* reporter at different temperatures

**Figure 1:**
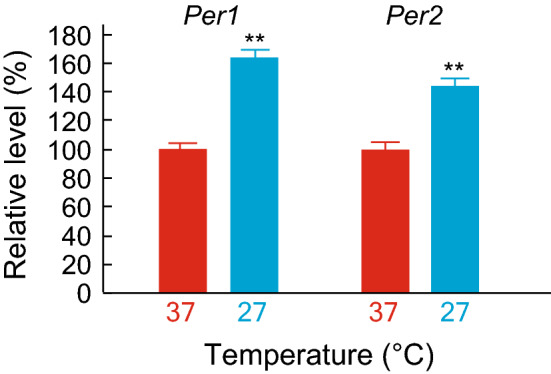
Effects of temperature on transcript levels of clock genes in Rat-1 fibroblasts. Relative mRNA level of *Per1* or *Per2* after 2-day culture at 37 °C or 27 °C*.* Mean with s.e.m. from 3 independent samples are shown. The expression levels at 37 °C were set to 100 in vertical axis. ** *p* < 0.01 compared to the data of 37 °C (Student’s t-test).

**Figure 2:**
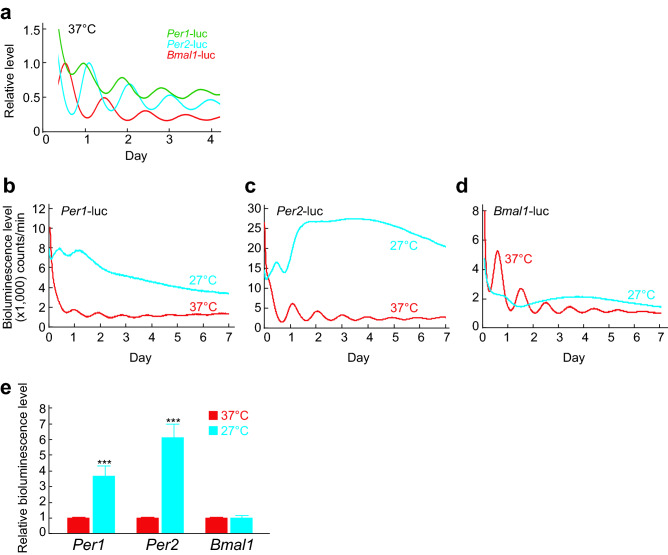
Effects of temperature on transcriptional reporters of clock genes. (**a**) Representative bioluminescence rhythms driven by *Per1*-luc, *Per2*-luc, or *Bmal1*-luc reporter. The first peak is normalized to 1 in the vertical axis. The rhythms were smoothing by 1-h centered moving average for clearness to compare the phase of each reporter. Representative data of bioluminescence rhythms of *Per1*-luc (**b**), *Per2*-luc (**c**), and *Bmal1*-luc (**d**) at 37 °C or 27 °C. (**e**) Relative bioluminescence levels of *Per1, Per2*, or *Bmal1* calculated by the area under the curve (AUC) from Day 0 to Day 3. The means of AUC at 37 °C were set to 1 in vertical axis. Mean with s.e.m. from 4 independent samples are shown. *** *p* < 0.001 compared to the data at 37 °C (Student’s t-test).

The previous studies revealed that expression levels of *Per1* and *Per2* are transiently induced by cytoplasmic Ca^2+^ increase both in the SCN and peripheral cells^18,19,22^. We found that temperature decrease chronically elevates cytoplasmic Ca^2+^ levels and *Per1* and *Per2* transcripts^18^ (Fig. 1). Based on the results, we thought that the cold responses of *Per1* and *Per2* genes could be monitored at transcriptional levels. We employed transcriptional reporters that express luciferase under the control of *Per1* or *Per2* promotor^22^. In addition, luciferase reporter of *Bmal1*^19^ promoter was also analyzed as a negative control reporter, because the mRNA levels of *Bmal1* showed no increase under lower temperature^18^. The cells were first synchronized with 1 h-pulse of 0.1 μM dexamethasone, and the medium was replaced with an air culture medium before measuring bioluminescence. In the mouse SCN and liver, *Per1* and *Per2* show expression rhythms that are anti-phasic to *Bmal1* expression rhythms^23^. Consistent with the in vivo expression, bioluminescence rhythms driven by the luciferase reporter of *Per1* or *Per2* promoter were nearly anti-phasic to the rhythms by *Bmal1* promotor in Rat-1 fibroblasts cultured at 37 °C (Fig. 2a). When the circadian rhythms were analyzed under 27 °C, we found that the clear bioluminescence rhythms driven by the all three reporters were severely damped (Fig. 2b–d). In addition, we found that the bioluminescence levels of *Per1* and *Per2* were markedly elevated. Then we analyzed bioluminescence levels by quantifying area under the curves (AUC) of the rhythms at both temperatures. *Per1* or *Per2* reporter showed 3.7-fold or 6.1-fold increase of the expression levels at 27 °C compared to 37 °C, respectively (Fig. 2e). On the other hand, the expression levels of *Bmal1* reporter at 27 °C showed no significant change compared to 37 °C, consistent with the mRNA levels reported in our previous study^18^. These results indicate that *Per1* and *Per2* luciferase reporters were applicable to monitor cellular response to the cold exposure.

### Ca^2+^-dependent transcription is activated by cold exposure

**Figure 3:**
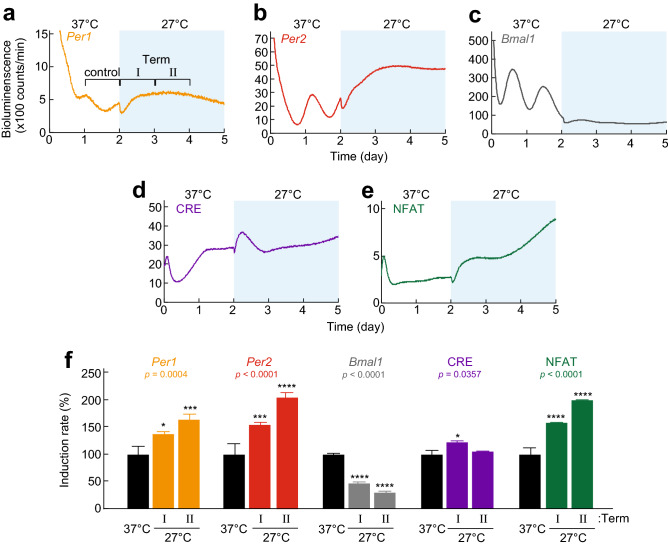
Effects of temperature shift on transcriptional reporters. Representative data of bioluminescence level of *Per1* (**a**), *Per2* (**b**), *Bmal1* (**c**), CRE (**d**), or NFAT (**e**) luciferase reporters in temperature shift experiment. The reporter cells were cultured at 37 °C for 2 days and then temperature was decreased to 27 °C during the rest of the experiment. (**f**) Effect of temperature decrease from 37 to 27 °C on bioluminescence signals of reporter cell lines. The induction rate was calculated by dividing the AUC during Term I or Term II (27 °C) by the AUC during day 1 to 2 (control, 37 °C) (marked as example in panel **a**). The means of induction rate at 37 °C was set to 100 in vertical axis. Mean with s.e.m. from 4, 8, 8, 4 or 3 independent samples for *Per1*, *Per2*, *Bmal1*, CRE, or NFAT luciferase reporter cells are shown, respectively. *p* values: one-way ANOVA among induction rate in Term I, II and control. * *p* < 0.05, ** *p* < 0.01, *** *p* < 0.001, **** *p* < 0.0001 compared to the data at 37 °C (Dunnett’s test).

The significant cold induction of bioluminescence levels driven by the *Per1* and *Per2* transcriptional reporters prompted us to investigate acute responses of the reporters to temperature shift from 37 to 27 °C in detail (Fig. 3). The cells were first cultured at 37 °C for 2 days, and then kept at 27 °C in the same luminescence detector for 3 days. For the control, the bioluminescence signals were monitored at 37 °C for 5 days (Fig. S1). We found that the bioluminescence rhythms driven by the *Per1*, *Per2* and *Bmal1* reporters observed at 37 °C was acutely damped at 27 °C (Fig. 3a–c), similar to the results of constant condition at 27 °C (Fig. 2). We analyzed effects of temperature on bioluminescence levels by calculating the AUC during Term I and II (as depicted in Fig. 3a). We found that the AUC of *Per1* reporter increased to 137% or 162% in Term I or II compared to control area of 37 °C, respectively (Fig. 3a and f). Similarly, the AUC of *Per2* reporter was increased to 153% (Term I) or 204% (Term II), whereas that of *Bmal1* reporter was decreased to 46% (Term I) or 29% (Term II) (Fig. 3b,c,f). These results indicate that the transcriptional reporters of *Per1* and *Per2* can be used to monitor temporal responses to the temperature shift.

We next investigated whether the cold response was observed in a Ca^2+^-dependent transcriptional reporter including Ca^2+^/cAMP response elements (CRE) or nuclear factor of activated T cell (NFAT)-binding elements. We found that temperature shift from 37 to 27 °C increased bioluminescence levels of the CRE reporter only during Term I, suggesting that the CRE reporter shows a transient response to the cold exposure (Fig. 3d and f). In contrast, the NFAT reporter, which reflects activation of Ca^2+^/calcineurin-NFAT signaling, showed a constant increase of the AUC in both Term I and II (Fig. 3e and f). These results demonstrate that CRE and NFAT-dependent transcriptional reporters are applicable to monitor cold Ca^2+^ signaling in addition to the *Per1* and *Per2* transcriptional reporters.

### Effects of benzyloxyphenyl NCX inhibitors on cold *Per2* induction

**Figure 4:**
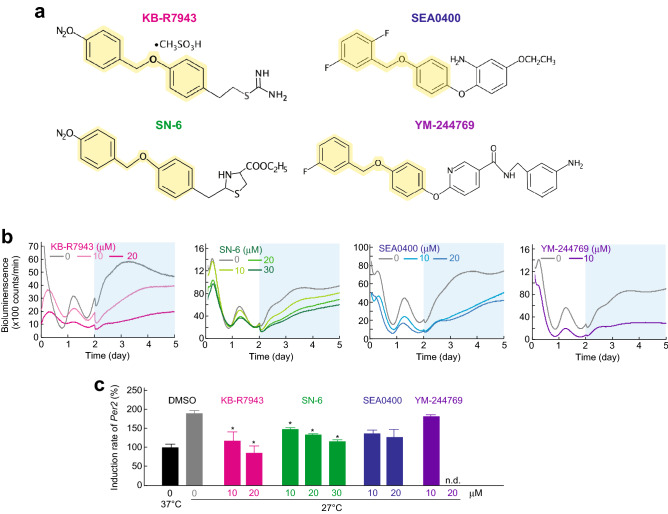
Effects of NCX inhibitors on cold *Per2* induction. (**a**) Structures of NCX inhibitors, KB-R7943, SN-6, SEA0400, and YM-244769. Yellow background shows a common structure of the four inhibitors. (**b**) Effects of NCX inhibitors on cold induction of *Per2*-luc reporter. (**c**) Effects of NCX inhibitors on induction rate of *Per2*-luc reporter. The induction rate was calculated by dividing the AUC during Term II (27 °C) by the AUC during day 1 to 2 (control, 37 °C) (marked as example in Fig. 3a). Because 20 µM YM-244769 showed cell toxicity, the data of that was not determined (n.d.). The means of induction rate of DMSO control group at 37 °C was set to 100 in vertical axis. Mean with s.e.m. from 4 independent samples are shown. * *p* < 0.05 compared to the DMSO control at 27 °C (Dunnett's test).

Based on the results of cold responses observed in the transcriptional reporter cell lines, we thought that the system might be useful to evaluate effects of chemical inhibitors on NCX-dependent cold Ca^2+^ signaling. First, we tested effects of small molecule NCX inhibitor KB-R7943^24^ (Fig. 4a), which blocks cold induction of cytoplasmic Ca^2+^ and *Per2* mRNA levels^18^. We found that KB-R7943 blocked the cold induction of bioluminescence levels of *Per2* reporter in a dose-dependent manner (Fig. 4b and c). In addition, we investigated the effect of KB-R7943 in cold induction by using Rat-1 *Per1*-luc reporter cells. Consistent with the results observed in *Per2*-luc cells, KB-R7943 dose-dependently inhibited the cold induction of *Per1* (Fig. S2a and b). Next, we evaluated other benzyloxyphenyl NCX inhibitors SN-6, SEA0400 and YM-244769^25–27^(Fig. 4a). SN-6 dose-dependently inhibited the cold induction of *Per2* similar to KB-R7943 (Fig. 4b and c). On the other hand, SEA0400 and YM-244769 showed no significant inhibitory effects on the *Per2* induction (Fig. 4b and c). Because KB-R7943 and SN-6 have relatively small functional groups compared to SEA0400 and YM-244769 (Fig. 4a), it is possible that small functional groups on benzyloxyphenyl skeleton are important for the pharmacological activity on the cold induction of *Per2* reporter.

### Correlation analysis of *Per2* induction and circadian rhythm parameters

**Figure 5:**
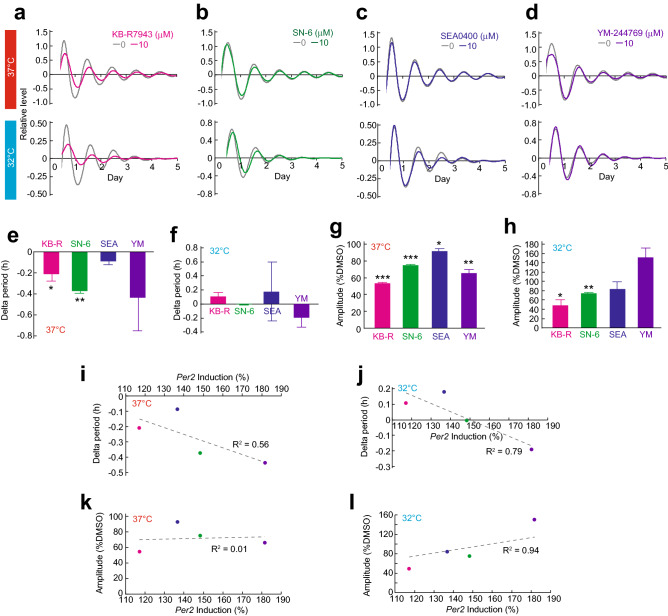
Correlation analysis between cold *Per2* induction and circadian rhythm parameters. Representative data of the effect of KB-R7943 (**a**), SN-6 (**b**), SEA0400 (**c**), or YM-244769 (**d**) on relative bioluminescence rhythms of Rat-1 *Bmal1*-luc cells. Effects of 4 NCX inhibitors on period length of bioluminescence rhythms at 37 °C (**e**) or 32 °C (**f**). Effects of 4 NCX inhibitors on amplitude at 37 °C (**g**) or 32 °C (**h**). Correlation analysis of cold *Per2* induction and period length at 37 °C (**i**) or 32 °C (**j**). Correlation analysis of cold *Per2* induction and amplitude at 37 °C (**k**) or 32 °C (**l**). Mean with s.e.m. from 4 independent samples are shown (**e**–**h**). * *p* < 0.05, ** *p* < 0.01, *** *p* < 0.001 compared to the DMSO control (Student’s t-test, with Bonferroni correction). Mean from 4 independent samples are shown (**i**–**l**).

In order to understand pharmacological actions of the four different benzyloxyphenyl NCX inhibitors on circadian rhythms, we accessed bioluminescence rhythms of Rat-1 *Bmal1*-luc cells cultured at 32 °C or 37 °C with or without 10 µM NCX inhibitors (Fig. 5a–d and Fig. S3). When the effects of the inhibitors on period length at 37 °C were analyzed, KB-R7943 and SN-6 significantly shortened the period compared to DMSO control group (Fig. 5e). On the other hand, at 32 °C, all the inhibitors showed no significant effect on the period (Fig. 5f). Next, we analyzed the effects of the inhibitors on amplitude of the bioluminescence rhythms. We found that all the inhibitors significantly decreased the amplitude of the circadian rhythms at 37 °C (Fig. 5g). On the other hand, at 32 °C, only KB-R7943 and SN-6 reduced the amplitude (Fig. 5h). These results demonstrate that KB-R7943 and SN-6 have strong effects especially at lower temperature.

Finally, we analyzed the relationships between the circadian rhythm parameters (Fig. 5a–h) and the cold *Per2* induction (Fig. 4). Correlation analysis demonstrated that effects on the period length showed a correlation with the cold induction of *Per2* (Fig. 5i and j). Importantly, effects on amplitude at 32 °C showed strong correlation with the cold induction of *Per2*, whereas effects on amplitude at 37 °C showed no correlation (Fig. 5k and l). Previously, we identified that the NCX-dependent Ca^2+^ signaling is activated at low temperature to increase amplitude of the cellular rhythms^18^. The inhibitory effects of NCX inhibitors on cold-*Per2* induction was strongly correlated with the effects on amplitude of the cellular rhythms especially at lower temperature (Fig. 5i), suggesting that the cold *Per2* induction reflects magnitude of the cold Ca^2+^ response in cells. In conclusion, the real-time monitoring of cold-*Per2* induction can be used as a system for detecting cold Ca^2+^ response.

## Discussion

**Figure 6:**
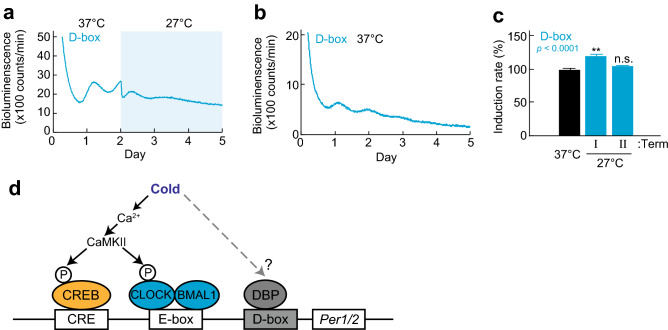
Effect of temperature shift on D-box reporter. (**a**) Representative bioluminescence rhythm of D-box luciferase reporter in temperature shift experiment. (**b**) Representative rhythms of D-box reporter cell line at 37 °C. (**c**) Induction rate of D-box reporter cell line. Mean with s.e.m. from 8 independent samples are shown. *p* values: one-way ANOVA among induction rate in Term I, II and control. ** *p* < 0.01 compared to the data at 37 °C (Dunnett’s test) (**d**) Schematic figure of transcriptional regulation of *Per1/2.* Transcription of *Per1/2* is regulated by cAMP response element (CRE), E-box (only one is shown here), and D-box. CRE is essential for various signaling pathways such as cAMP, Ca^2+^ and Ras, while E-box is the target of CLOCK/BMAL1 regulation, and D-box are regulated by E4BP4 and DBP.

Ca^2+^ signaling have been intensively studied in input pathway of the circadian clock^28,29^. In the SCN, light signals are transmitted by glutamate which activates NMDA receptor to promote Ca^2+^ influx^30^. The intracellular Ca^2+^ activates MAPK and CaMKII to phosphorylate transcriptional factor CREB. Because promotor regions of *Per1* and *Per2* contain CRE^22,28,29^, *Per1* and *Per2* are increased immediately after light exposure during subjective night in the SCN^31–33^*.* Consistent with the photic response, we found that the CRE-dependent transcription is transiently activated by temperature shift from 37 to 27 °C in Rat-1 fibroblasts. In contrast, the *Per1* or *Per2* reporter showed continuous increase after the cold exposure (Fig. 3). The promoter regions of *Per1* and *Per2* contain E-box and D-box sequences^34^, in addition to the CRE. We found that D-box reporter also showed a transient increase of bioluminescence level in response to the temperature decrease (Fig. 6a–c). It is possible that cooperative actions of CRE, E-box and D-box are important for the long-term response of *Per1* and *Per2* to the cold exposure (Fig. 6d).

Cold-Ca^2+^ signaling was originally found in studies of cold tolerance in plants^35–37^. Interestingly, we found that the cold Ca^2+^ signaling is conserved among bacteria, plants, insects and mammals, indicating that the Ca^2+^ signal is an ancestral mechanism for cold response. Because the intracellular Ca^2+^ levels continuously increased during cold exposure in mammalian cells^18^, the cold Ca^2+^ signal is unique compared to general Ca^2+^ signaling that shows a transient increase. Therefore, we think that long term investigation of the cold-Ca^2+^ signaling in living cells is very important for understanding of fundamental temperature responses. In this study, we attempted to monitor cold Ca^2+^-dependent transcription by using *Per2*-luc stable expression cell line. By using this system, we investigated the efficacies of NCX inhibitors. Benzyloxyphenyl NCX inhibitors are thought to interact with a specific receptor site in α-2 loop of NCX1 proteins to block ion transport pore(s)^38^. KB-R7943 or SN-6, which showed strong effects on *Per2* response, has a smaller functional group on the benzene ring compared to SEA-0400 and YM-244769 (Fig. 4a). Therefore, it is possible that bulky functional groups on the benzene ring in SEA-0400 and YM-244769 interfere access of the inhibitor to the putative receptor sites in NCX proteins. In conclusion, we believe that this system can be a powerful tool for investigating temperature response in living cells.

## Methods

### RT-qPCR experiments by using Rat-1 fibroblasts

The Rat-1 fibroblasts were purchased from American Type Culture Collection (ATCC). The cells were plated on 35-mm dishes (1.0 × 10^6^ cells/dish) and cultured at 37 °C under 5% CO_2_ in a culture medium [DMEM (Sigma-Aldrich, catalog no. 5796) supplemented with 10% FBS (Biosera), 50 U/ml penicillin and 50 μg/ml streptomycin]. One day after the plating, the medium was changed to air culture medium [DMEM (Sigma-Aldrich, catalog no. D2902) supplemented with 10% FBS, 3.5 mg/ml glucose, 25 U/ml penicillin, 25 μg/ml streptomycin and 10 mM HEPES–NaOH (pH 7.0)], and the cells were cultured at 37 °C or 27 °C under air. The cells were collected on day 2 by using 600 μl TRIzol (Invitrogen). Total RNA was prepared from cultured cells using RNeasy Kit (Qiagen) according to the manufacturer’s protocol. RT-qPCR analysis was performed as described previously^18,39^.

### Real-time monitoring of gene expression rhythms in mammalian cells

Real-time monitoring of gene expression rhythms in mammalian cells was performed by using Rat-1 fibroblasts stably expressing luciferase driven by *Per1* promoter*, Per2* promoter^22^*, Bmal1* promoter^19^, D-box elements^34^, CRE or NFAT binding elements. The CRE reporter or NFAT reporter was constructed by inserting 4 repeats of CREB-binding sequences (AGCC**TGACGTCA**GAG) or 4 repeats of NFAT-binding sequences (GGA**GGAAAAACTGTTTCA**TACAGAAGGCGT) into pGL3-Basic vector (Promega). The fibroblasts were plated on 35-mm dishes (1.0 × 10^6^ cells/dish) and cultured at 37 °C under 5% CO_2_ in the culture medium [DMEM (Sigma-Aldrich, catalog no. 5796) supplemented with 10% FBS (Biosera), 50 U/ml penicillin and 50 μg/ml streptomycin]. One day after the plating, the cells were treated with 0.1 μM dexamethasone for 1 h, and the medium was replaced with the air culture medium [DMEM (Sigma-Aldrich, catalog no. D2902) supplemented with 10% FBS, 3.5 mg/ml glucose, 25 U/ml penicillin, 25 μg/ml streptomycin, 0.1 mM luciferin and 10 mM HEPES–NaOH (pH 7.0)]. The bioluminescence signals were continually recorded from the cells cultured under air in a dish-type bioluminescence detector, LumiCycle (Actimetrics). In the temperature shift experiment, the temperature was first set to 37 °C for 2 days and then set to 27 °C for the rest of the measurement.

For normalization of dish-to-dish variation of the bioluminescence levels, the raw data were divided by mean bioluminescence signals recorded for 6 days. The normalized rhythms were detrended by subtracting 24-h centered moving averages, and the average of second, third and fourth peak and trough were used for calculating the amplitudes of the rhythms. Period length was calculated using the average value of peak-to-peak periods and trough-to-trough periods one day after the dexamethasone treatment of cultured cells.

## Supplementary Information

Supplementary Information.

## Data Availability

The datasets generated during and/or analyzed during the current study are available from the corresponding author on reasonable request.
