# Supplementary material for: Real time monitoring of cold Ca2+ dependent transcription and its modulation by NCX inhibitors
Source: Sci Rep. 2022 Oct 15;12:17325. doi: 10.1038/s41598-022-22166-4 (PMC9569354; doi:10.1038/s41598-022-22166-4)
Supplement: Supplementary file 1 — Supplementary Information. [file 41598_2022_22166_MOESM1_ESM.pdf]

Supplementary Materials for
Real time monitoring of cold Ca^{2+} dependent transcription and its modulation by NCX inhibitors

Hsin-tzu Wang, Shiori Miyairi, Miho Kitamura, Kosuke Iizuka, Yoshimasa Asano, Takashi Yoshimura, Naohiro Kon*

*Corresponding author. Email: kon.naohiro@itbm.nagoya-u.ac.jp

This PDF file includes:

Fig. S1 to S3
Figure legend for Fig. S1-S3

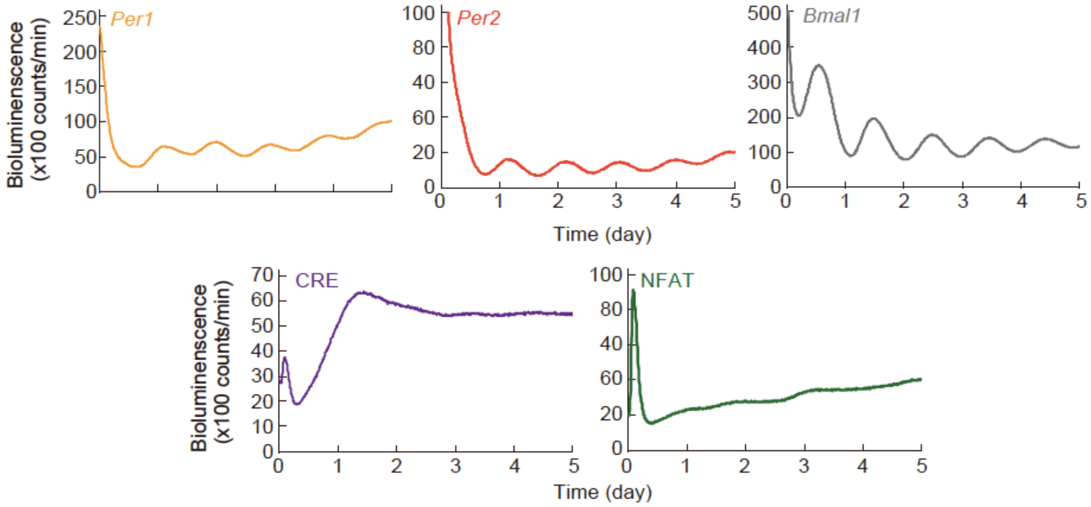

Figure S1. Monitoring of bioluminescence signals from *Per1*, *Per2*, *Bmal1*, CRE or NFAT luciferase reporter cell lines. Representative data of bioluminescence level of each reporter cell line at 37°C.

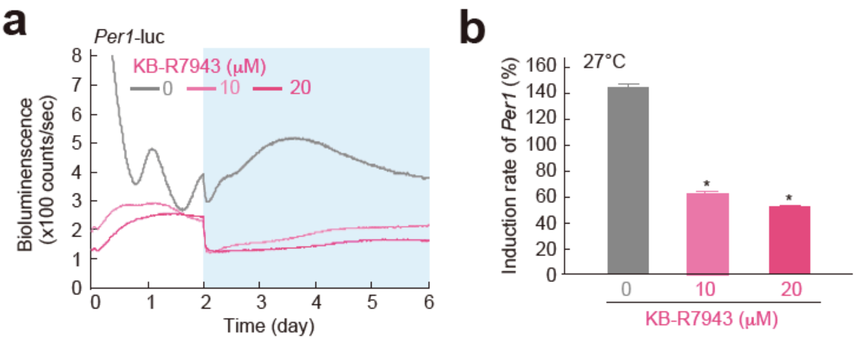

Figure S2. Effects of NCX inhibitor on cold *Per1* induction. (**a**) Effects of 10 μM or 20 μM NCX inhibitor KB-R7943 on cold induction of Rat-1 *Per1*-luc cells. (**b**) Effects of KB-R7943 on induction rate of *Per1*-luc reporter. The induction rate was calculated by dividing the AUC during Term II (27°C) by the AUC during day 1 to 2 (control, 37°C) (marked as example in Fig. 3a). Mean with s.e.m. from 3 independent samples are shown. * $p < 0.05$ compared to the DMSO control at 27°C (Dunnett's test).

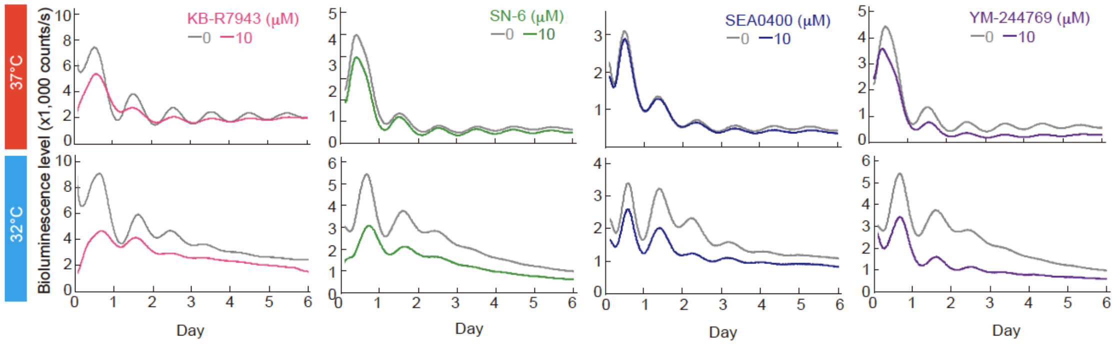

Figure S3. Effect of NCX inhibitors on rhythms of Rat-1 *Bmal1*-luciferase cell lines. Representative data of bioluminescence level of the Rat-1 *Bmal1*-luc cells. The cells are treated with 10 μM KB-R7943, SN-6, SEA0400, or YM-244769, respectively.
